# Speech in the foreign accent syndrome: differential diagnosis between organic and functional cases

**DOI:** 10.1590/1980-57642020dn14-030015

**Published:** 2020

**Authors:** Simone dos Santos Barreto, Karin Zazo Ortiz

**Affiliations:** 1 Departamento de Formação Específica em Fonoaudiologia, Instituto de Saúde de Nova Friburgo, Universidade Federal Fluminense - Nova Friburgo, RJ, Brazil.; 2 Departamento de Fonoaudiologia, Escola Paulista de Medicina, Universidade Federal de São Paulo - São Paulo, SP, Brazil.

**Keywords:** speech disorders, speech production measurement, symptom assessment, diagnosis, differential, distúrbios da fala, medida da produção da fala, avaliação de sintomas, diagnóstico diferencial

## Abstract

Foreign accent syndrome (FAS) is an extremely rare disorder, with 112 cases described until 2019. We compare two cases of the foreign accent syndrome in native speakers of Brazilian Portuguese in its classic form (FAS) and psychiatric variant (FALS). Two cases were analyzed: (1) a right-handed, 69-year-old man, with a prior history of stroke, and (2) a right-handed, 43-year-old woman, diagnosed with schizophrenia. They were evaluate for language and speech, including the speech intelligibility. Both patients had speech impairments complaints, similar to a new accent, without previous exposure to a foreign language. However, the onset of the speech disorder was sudden in case 1 and insidious and with transient events in case 2, with speech intelligibility scores of 95.5 and 55.3% respectively. Besides neurologic impairment, the clinical presentation of FALS was extremely severe and differed to that expected in FAS cases, in which speech intelligibility is preserved.

## RESUMO.

A síndrome do sotaque estrangeiro (SSE) é um distúrbio extremamente raro, com 112 casos descritos até 2019. Foram comparados dois casos de SSE em falantes nativos do português brasileiro em sua forma clássica e em sua variante psiquiátrica. Os seguintes casos foram analisados: (1) homem de 69 anos, destro, com história prévia de acidente vascular cerebral, e (2) mulher de 43 anos, destra, com diagnóstico de esquizofrenia. Eles foram avaliados quanto à linguagem e à fala, incluindo a inteligibilidade. Ambos apresentaram alterações de fala, como um sotaque novo adquirido, sem exposição prévia à língua estrangeira. Entretanto, o início do distúrbio foi súbito no caso 1 e insidioso e com eventos transitórios no caso 2, com escores de inteligibilidade da fala de 95,5 e 55,3%, respectivamente. Além do comprometimento neurológico, a apresentação clínica da variante psiquiátrica foi mais grave e diferiu dos casos de SSE, cuja inteligibilidade da fala é preservada.

## INTRODUCTION

Foreign accent syndrome (FAS) is an extremely rare disorder characterized by the appearance of speech changes, perceived by the speaker and listeners as an acquired foreign accent.^1^ Most cases occur after a brain damage, however there are cases associated with psychological or psychiatric conditions, which are classified as functional^2^ or psychogenic,^3^ and in this case they are called foreign accent-like syndrome (FALS).^4^ Differentiating these conditions is a challenge for the clinicians.^3^

In a recent review published in 2019, Marien et al. identified 112 described cases since 1907, when the first case was related by Pierre Marie.^2^ Most of cases involved adults, female, right-handed, native English speakers who had no previous contact with the acquired accent. More than 70% of the cases were organic-neurogenic type, resulting from stroke and mostly associated with supratentorial lesion in the left hemisphere. FAS rarely occurs in isolation, with mutism occurring in more than 40% post-stroke in the acute phase, followed by aphasia, dysarthria, apraxia of speech, agraphia and alexia, and the remission of FAS is observed in 20% of cases. The functional type of FAS covered 16.1% of the cases described, with remission observed in 38% of them.

In Brazil, 7 cases were published.^5^ ^,^ ^6^ ^,^ ^7^ ^,^ ^8^ ^,^ ^9^ ^,^ ^10^ Given the scarcity of studies in the area, clinical description of the communication disorder of such cases can help the professionals working in the neuropsychological field to care out the differential diagnosis and enable the appropriate follow-up of each case.

The objective of this study was to compare two cases of the syndrome in native speakers of Brazilian Portuguese in its classic form (FAS) and psychiatric variant (FALS).

## METHODS

This study was approved by Research Ethics Committee of the Universidade Federal de São Paulo (N. 3.584.372), and the participants signed the Informed Consent Term.

Two patients with acquired speech disorders followed up at the Speech, Language and Hearing Sciences Department of the University, who were native speakers of Brazilian Portuguese, took part in this observational study. Both patient reported never been in contact with other languages than Brazilian Portuguese.

- case 1: a right-handed, 69-year-old man with a prior history of stroke and imaging scan disclosing ischemic lesion in the left parietal lobe due to a cardioembolic mechanism;^11^ in follow up with a cardiologist and a neurologist;
- case 2: a right-handed, 43-year-old woman, diagnosed with schizophrenia, with imaging scan disclosed multiple periventricular and subcortical ischemic foci in the small-vessels zones due to microangiopathy; the patient was followed by a psychiatrist, who prescribed a medication.

The patients were submitted to a speech-language assessment entailing interview, Boston Diagnostic Aphasia Examination^12^ ^,^ ^13^ - for language assessment, and orofacial and speech apraxia assessment protocols.^14^ The interview was conducted with the patient and his brother in case 1, and only with the patient in case 2. The tests were applied and analyzed according to their norms.^12^ ^,^ ^13^ ^,^ ^14^

An auditory-perceptual analysis based on speech samples was also carried out. Data from speech samples were recorded during the reading of a list of words of varying lengths (Appendix 1), a text from Dysarthria protocol,^15^ and a spontaneous speech sample, obtained from the Boston Cookie Theft description.

Two speech therapists with experience in the area of acquired neurological disorders independently performed the phonetic transcription of the recorded speech samples which were analyzed for segmental (phonemes and syllables) and suprasegmental (pauses, intonation and stress on words and/or phrases) aspects of speech production. The identified errors were classified into segmental errors (syllable or phoneme omissions, additions or substitutions and morphemic changes) and suprasegmental errors (atypical pauses for respiratory reload, inappropriate accent, inadequate word stress and excessive pitch variations). Only errors identified by both evaluators were considered. The frequency distribution of errors was determined according to error type (segmental or suprasegmental) and speech sample type analyzed.

Speech intelligibility was measured as the percentage of correctly transcribed words of the total words produced from the spontaneous speech sample by each evaluator. The mean of the scores of the two examiners was calculated. The perceptual assessment of an accent was carried out by 10 raters blinded to patient diagnosis, using a Likert scale, as suggested in a previous study.^16^ The scale ranged from 0-4, in which 0 indicated definitely a native speaker of Brazilian Portuguese, and 4 definitely a foreigner speaking this language. Twelve edited sentences from the text reading task formed the speech sample. Each group of 5 raters was presented with 6 different sentences produced by each of the two patients. After listening to each speech stimulus once, the listeners independently rated them for the presence of an accent. The median of ratings attributed by the listeners for all 12 stimuli produced by each speaker was calculated. The listeners also identified which accent they perceived in each case.

The data were analyzed descriptively using absolute and relative frequency.

## RESULTS

Both patients had complaints of speech impairment, similar to a new accent, without previous exposure to a foreign language as reported by them. In case 1, the accent change was also reported by the patient's brother. However, the onset of the speech disorder was sudden in case 1 and insidious and characterized by transient events in case 2. In the latter case, the episodes of speech changes were sporadic and associated with appendicular muscle spasms, triggered by situations of emotional stress, and assumed the characteristic of apparent accent only after six years.

With regard to the formal assessment of speech and language, mild verbal and non-verbal apraxia were found only in case 1, with score of 131 in orofacial praxis and the occurrence of hesitations, reiterations and repetitions of initial syllables and words in the Apraxia protocol. Despite articulation errors and prosodic abnormalities, neither of the patients had a diagnosis of dysarthria. In addition, the patients performed satisfactorily on the language tests.

The results of the perceptive-auditory analysis in terms of frequency of error types in the two cases are shown in Table 1.

**Table 1.** Frequency of error types in foreign accent syndrome and foreign accent-like syndrome cases.

Error types	Reading words				Reading text				Spontaneous speech			
	Case 1		Case 2		Case 1		Case 2		Case 1		Case 2	
	n	%	n	%	n	%	n	%	n	%	n	%
Segmentals												
Syllable addition	0	0	1	4	0	0	0	0	0	0	0	0
Syllable omission	0	0	2	7	1	14	2	7	2	25	3	6
Phoneme addition	0	0	2	7	1	14	1	3	0	0	2	4
Phoneme omission	0	0	12	41	5	72	9	30	5	62,5	22	44
Phoneme substitution	4	100	12	41	0	0	17	57	0	0	23	46
Morphemic errors	0	0	0	0	0	0	1	3	1	12,5	0	0
Total errors	4	100	29	100	7	100	30	100	8	100	50	100
Suprasegmentals												
Atypical pauses (syllables)	0	0	0	0	0	0	0	0	0	0	0	0
Atypical pauses (words)	-	-	-	-	0	0	0	0	0	0	3	50
Inappropriate accent	0	0	1	100	2	100	0	0	3	100	0	0
Inadequate word stress	-	-	-	-	0	0	0	0	0	0	0	0
Excessive pitch variations	-	-	-	-	0	0	0	0	0	0	3	50
Total errors	0	0	1	100	2	100	0	0	3	100	6	100

The intelligibility of the spontaneous speech sample was 95.5% in case 1 and 55.3% in case 2.

In relation to the perceptual assessment of an accent, the median score for the first case was 1 (almost certain that patient is a native speaker of Brazilian Portuguese) and the second case was 4 (definitely a foreigner speaking Brazilian Portuguese). Regarding the perceived accent, 6/10 listeners identified a European Portuguese accent in case 1, while 7/10 listeners attributed a French accent in case 2.

## DISCUSSION

 The main features of this syndrome were identified in both of the cases reported: perceived foreign accent by the speakers and listeners; and the absence of previous exposure to the language whose accent was apparently acquired.^16^ ^,^ ^17^ Regarding imaging diagnosis, despite the abnormalities apparent in case 2, their location differed to other cases of FAS reported, whose lesions were predominantly located in anterior areas of the dominant hemisphere and adjacent to the precentral sulcus and anterior insula^3^ ^,^ ^18^ and parietal lobel,^2^ as in case 1.

Segmental and suprasegmental deficits were detected, although the former predominated in both cases. The higher occurrence of phonemic substitutions and omissions in the two cases corroborate the findings of other reported cases.^16^ ^,^ ^17^ However, analysis of the frequency distribution of these deficits revealed that the segmental errors were more frequent and less influenced by the type of speech sample analyzed in case 2. In addition, a broader variety of prosodic deficits was identified in the spontaneous speech of the second patient. Greater variability and inconsistency in the errors observed in case 2 reinforce the hypothesis of a functional condition,^3^ as well as the absence of associated neurogenic speech and/or language disorders.^2^ ^,^ ^3^

The results of perceptual assessment by the listeners, which attributed a higher score to case 2, are consistent with the higher occurrence of segmental and suprasegmental errors. These errors make the speech of this individual more closely resemble that of a foreign speaker and have a marked effect on intelligibility. This characteristic differs to the organic-neurogenic cases of FAS, in which the preservation of speech intelligibility is one of the defining features of this type of speech disorder.^18^

Based on these results, it was concluded that, besides neurologic impairment, the late clinical presentation of FALS was extremely severe and differed to that expected in FAS cases, in which speech intelligibility is preserved.^19^ Moreover, the changes seen in FAS are not intermittent and associated spasms are not expected. These features can help distinguish FALS.

## Appendix 1. List of words.

1. Cão

2. Sol

3. Ar

4. Os

5. Te

6. Na

7. Filé

8. Sofá

9. Amor

10. Fome

11. Raiva

12. Cama

13. Parabéns

14. Guaraná

15. Caneta

16. Saudade

17. Ônibus

18. Mérito

19. Abacaxi

20. Opinião

21. Felicidade

22. Geladeira

23. Específico

24. Fotógrafo
